# Exploring the role of gut microbiota in host feeding behavior among breeds in swine

**DOI:** 10.1186/s12866-021-02409-6

**Published:** 2022-01-03

**Authors:** Yuqing He, Francesco Tiezzi, Jeremy Howard, Yijian Huang, Kent Gray, Christian Maltecca

**Affiliations:** 1 Department of Animal Science, North Carolina State University, 120 W Broughton Dr, Raleigh, 27607 NC USA; 2 Department of Agriculture, Food, Environment and Forestry, University of Florence, Piazzale delle Cascine 18, 50144 Firenze, Italy; 3 Smithfield Premium Genetics, Rose Hill, 28458 NC USA

**Keywords:** Swine, Gut microbiota, Feeding behavior, Breed, Growth stage

## Abstract

**Background:**

The interplay between the gut microbiota and feeding behavior has consequences for host metabolism and health. The present study aimed to explore gut microbiota overall influence on feeding behavior traits and to identify specific microbes associated with the traits in three commercial swine breeds at three growth stages. Feeding behavior measures were obtained from 651 pigs of three breeds (Duroc, Landrace, and Large White) from an average 73 to 163 days of age. Seven feeding behavior traits covered the information of feed intake, feeder occupation time, feeding rate, and the number of visits to the feeder. Rectal swabs were collected from each pig at 73 ± 3, 123 ± 4, and 158 ± 4 days of age. DNA was extracted and subjected to 16 S rRNA gene sequencing.

**Results:**

Differences in feeding behavior traits among breeds during each period were found. The proportion of phenotypic variances of feeding behavior explained by the gut microbial composition was small to moderate (ranged from 0.09 to 0.31). A total of 21, 10, and 35 amplicon sequence variants were found to be significantly (q-value < 0.05) associated with feeding behavior traits for Duroc, Landrace, and Large White across the three sampling time points. The identified amplicon sequence variants were annotated to five phyla, with *Firmicutes* being the most abundant. Those amplicon sequence variants were assigned to 28 genera, mainly including *Christensenellaceae*_R-7_group, *Ruminococcaceae*_UCG-004, *Dorea, Ruminococcaceae*_UCG-014, and *Marvinbryantia*.

**Conclusions:**

This study demonstrated the importance of the gut microbial composition in interacting with the host feeding behavior and identified multiple archaea and bacteria associated with feeding behavior measures in pigs from either Duroc, Landrace, or Large White breeds at three growth stages. Our study provides insight into the interaction between gut microbiota and feeding behavior and highlights the genetic background and age effects in swine microbial studies.

**Supplementary Information:**

The online version contains supplementary material available at 10.1186/s12866-021-02409-6.

## Background

Feeding behavior is a collection of activities that reflect the feelings of hunger or satiety of animals at different physiological or developmental stages [1]. The feeding behavior of pigs can be evaluated by feed intake, time spent eating, number of meals per day, length of and interval between meals, and feeding rate [2, 3]. Numerous studies have addressed the significant relationship between feeding behavior and growth performance [4], carcass quality [4], and feed efficiency [5, 6] in swine. Feeding behavior conveys essential information for producers to optimize feed management and identify sick animals to achieve production goals.

The regulation of feeding behavior results from a vast network of interactions between the gastrointestinal (GI) tract and the brain. Miroschnikow et al. [7] found evidence of gut-derived chemical or neural signals functioning on the central nervous system to regulate various physiological activities, including feeding behavior. The diverse microorganisms colonizing the GI tract (hereafter referred to as gut microbiota) are extensively involved in nutrient absorption, energy metabolism, immune activities, and disease resistance through profound symbiotic interactions with the host in humans and animals [8, 9]. In human and rodent models, studies suggest that the gut microbiota modifies brain function and modulates appetite and eating behavior [1, 10, 11]. Meanwhile, diet and feeding patterns have been found to induce rapid shifts in the gut microbial composition [12, 13]. This intimate relationship between the gut microbiota and host feeding behavior relies on energy exchange. The microbiota relies on the host for nutrition to maintain its growth and population, while the host benefits from the metabolic activity of microbiota, partly digesting and assimilating the produced metabolites [1].

Host genetic background has been found to influence both feeding behavior [14] and gut microbiota composition [15] in pigs. Additionally, both feeding behavior [2] and gut microbial community [16–18] are dynamic over time. The gut microbiota matures with age and relatively stabilizes in adults, with the main changes occurring during the post-natal and peri-weaning periods, which are mainly associated with maternal, nutritional, and environmental factors [16, 19–21]. Longitudinal investigations in pigs have revealed several enterotypes of the gut microbiota and their relationships with growth performance at different stages during the growing-finishing phase [17, 22]. These findings indicate that the interaction between the gut microbiota and host feeding behavior may depend on factors such as host genetics and age.

However, limited information on this interaction is available in different breeds of pigs during the growing-finishing period. Our previous study characterized differences in the gut microbial composition and investigated its associations with feed efficiency at three time points across the three breeds [23]. Based on that, this study aims (i) to compare feeding behavior traits among three commercial breeds; (ii) to investigate the role of the gut microbiota in explaining the variations of phenotypic traits; (iii) to identify the specific microbes that are associated with feeding behavior at different stages during the growing-finishing stage in swine.

## Methods

### Experimental animals and sample collection

Data used in this study were provided by Smithfield Premium Genetics (SPG; Rose Hill, NC, USA). Procedures for rectal swab collection were approved by the Institutional Animal Care and Use Committee, North Carolina State University, and National Pork Board. Microbial and feed efficiency data from this experiment are described in details in [23] and will therefore not be discussed in the present study. This study included 651 boars of either Duroc (**DR**; n =205), Landrace (**LR**; n = 226), or Large White (**LW**; n = 220) breed. Boars were the progeny of the mating of 28 sires and 124 dams for DR, 27 sires and 154 dams for LR, and 45 sires and 161 dams for LW. The number of siblings per family ranged from one to five for DR and from one to three for LR and LW. Most of the families (84% for DR, 90% for LR, and 96% for LW) had one or two siblings. The distributions of the family relationships were similar across the three breeds. The family relationship among individuals is provided in Additional file (1) Pigs weaned at the mean age of 24 days were kept in single-breed groups and housed in pens with an average of nine, eleven, and ten pigs per pen (12.4 m^2^) for DR, LR, and LW, respectively. There were 64 such pens located across eight rooms (8 pens per room). Each room had all three breeds with headcounts as presented in Additional file (2) The experiment was conducted concurrently for three breeds in each room from May to December 2017. Each pen was installed with a single-space FIRE feeder (Osborne Industries Inc., Osborne, KS), which allows one pig to visit at a time. The feed consumption, feeder occupation time, bodyweight, and animal identifier were recorded whenever a pig visited the feeder. Pigs had 24-hour access to the feeder, and they were provided the same pelleted feed over the entire study. Details of the diet formulation are provided in Additional file (3) Feeding events were recorded on three breeds from 73 ± 3 to 163 ± 6 days of age. Rectal swabs were collected from each pig repeatedly at three time points (73 ± 3, 123 ± 4, and 158 ± 4 days of age).

### DNA extraction and 16 S rRNA gene sequencing

The procedures to extract DNA (gDNA) from each rectal swab for Illumina library preparation are described in Lu et al. [22]. After DNA extraction, 100 µL of crude DNA was purified using a QIAquick 96 PCR purification kit (Qiagen, MD, USA). Purification procedures were carried out according to the manufacturer’s protocol with a few minor modifications described in Lu et al. [22]. Purified DNA concentration was quantified using the Qubit 2.0 Fluorometer (Thermo Fisher Scientific Inc., MA, USA).

The V4 region of the 16 S rRNA gene from genomic DNA was amplified using the primers 515 F (5-GTGCCAGCMGCCGCGGTAA-3) and 806R (5-GGACTACHVGGGTWTCTAAT-3) to generate indexed libraries, as described in [22, 24]. Equal amounts of amplicon libraries quantified using the Qubit dsDNA assay kit (Thermo Fisher Scientific Inc., MA, USA) were pooled together. The pooled amplicons were purified using the Agencourt AMPure XP beads (Beckman Coulter Inc., IN, USA), following the manufacturer’s protocol. Purified pools were supplemented with 5–10% PhiX control DNA and were sequenced with Illumina MiSeq paired-end 2 × 250 bp + 13 bp index reactions using the 600v3 kit. Negative controls of microbial DNA-free water were included to confirm the absence of bacterial contamination during the Illumina library preparation. ATCC mock community standards (20-strain staggered mix, MSA-1003; ATCC, VA, USA) were used as positive controls to confirm the absence of lineage-specific biases in the upstream methods. Samples were sequenced at the DNA Sequencing Innovation Lab at the Center for Genome Sciences and Systems Biology at Washington University (St. Louis, MO, USA).

### Data editing

#### Phenotypic data

Data quality control was performed on the feeding events recorded by the FIRE feeder to identify and remove errors and outliers due to feeder malfunctions or animal-feeder interactions. The quality control categories and procedures on FIRE feeder data used in this study are described by Casey et al. [25]. After quality control, a total of 470,414 feeding records remained in the dataset. Corresponding to the three time points of rectal swab collection (T1: 73 ± 3 days of age; T2: 123 ± 4 days of age; T3: 158 ± 4 days of age), feeding records were split into three periods (P1: 73 ± 3 to 98 days of age; P2: 99 to 140 days of age; P3: 141 to 163 ± 6 days of age). The breakpoints at 98 and 140 days of age are the midpoints between T1 and T2 and T2 and T3 of rectal swab collection. For each period, seven feeding behavior measures, including the average daily feed intake (**ADFI**), average daily feeder occupation time (**AOTD**), average daily feeding rate (**ADFR**), average daily number of visits to the feeder (**ANVD**), average feed intake per visit (**AFIV**), average feeder occupation time per visit (**AOTV**), and average feeding rate per visit (**AFRV**), were calculated for each pig similar to what described by Lu et al. [6].

#### Sequencing data

The raw sequencing reads obtained from the Illumina platform were converted into fastq format files using MiSeq Reporter. Paired sequences were merged into a single sequencing file using FLASH (v1.2.11), with the minimum overlap length was set to 100 bp and the maximum length was set to 250 bp [26]. Sequences were trimmed to remove the primers and were demultiplexed using the Quantitative Insights Into Microbial Ecology (QIIME2) [27]. The amplicon sequence variant (**ASV**) feature table was constructed and denoised using the Divisive Amplicon Denoising Algorithms 2 (DADA2) with default settings and no truncation [28]. Taxonomic information was annotated using the Ribosomal Database Project (RDP) Classifier (v2.4) based on the SILVA reference database (v132) [29] with a confidence cutoff of 0.8. The ASVs were filtered by a minimum total count of 1000 across all the samples and were removed with a prevalence rate less than 0.05 at each sampling time point. A total of 750, 724, and 824 ASVs remained after the quality control for time point 1 (T1; 73 days of age), time point 2 (T2; 123 days of age), and time point 3 (T3; 158 days of age), respectively.

### Statistical analysis

#### Effects of breed on feeding behavior traits

Before analysis, phenotypes normality assumptions were verified, and traits ADFR during P1, AFRV during P1 and P2, and ANVD, AFIV, and AOTV during all three periods were log-transformed in the R environment (v4.0.2) [30]. To explore the relationship between feeding behavior traits, pairwise Spearman’s rank correlation coefficient (r_s_) was estimated for each breed group during each period due to the normality assumption’s violation in some traits [31]. Statistical significance for the correlations was considered present at α ≤ 0.05.

To estimate the effect of breed and to account for the environmental impact on feeding behavior traits, a linear mixed model was constructed using the PROC GLIMMIX in SAS (v9.4, SAS Institute, Carry, NC, USA). The model was conducted on each trait during each period separately, with Breed and Room fitted as the fixed effects and Sire and Pen fitted as the random effects. The least-squares means for fixed effects were obtained using the LSMEANS statement of the PROC GLIMMIX, and they were compared among breeds during each period. A P-value less than or equal to 0.05 was considered significantly different.

#### Microbial parameter of host feeding behavior traits

We applied the following two models to investigate the proportion of variance in host phenotypes due to differences in the gut microbial composition among individuals for each breed. Microbial measurements at T1 (start of P1), T2 (midpoint of P2), and T3 (end of P3) were used in the following models to estimate the proportion of variation in feeding behavior during the respective period. First, we defined a baseline model as:

$${y}_{ijkl}=\mu { + Room}_{i}+ {sire}_{j}+ {pen}_{k}+{e}_{ijkl}$$

where ${y}_{ijkl}$ is the feeding behavior trait, $\mu$ is the overall intercept, ${Room}_{i}$ is the fixed effect of *i*^th^ room, ${sire}_{j}$ is the random effect of *j*^th^ sire, ${pen}_{k}$ is the random effect of *k*^th^ pen, and ${e}_{ijkl}$ is the residual. The effects of sire, pen, and residual were assumed to be distributed as N(0, $\varvec{I}{{\upsigma }}_{p}^{2}$), N(0, $\varvec{I}{{\upsigma }}_{p}^{2}$), and N(0, $\varvec{I}{{\upsigma }}_{p}^{2}$), respectively; where **I** was an identity matrix, and ${{\upsigma }}_{2}^{2}$, ${{\upsigma }}_{p}^{2}$, and ${{\upsigma }}_{e}^{2}$ were the variances for the respective effects.

To estimate the variance in feeding behavior that is contributed by the gut microbiota of pigs at each growth stage, we then defined a univariate mixed model, in which microbial information was added to the baseline model as:

$${y}_{ijklm}=\mu { + Room}_{i}+ {sire}_{j}+ {pen}_{k}+ {m}_{l}+{e}_{ijklm}$$

where the model parameters are the same described in the baseline model, with the addition of ${m}_{l}$, which contains the random microbial effect for animal *l* at the given time point with m ~ N (0, $\varvec{M}{{\upsigma }}_{m}^{2}$), where $\varvec{M}$ is the microbial relationship matrix and ${{\upsigma }}_{m}^{2}$ is the microbial variance. The matrix $\varvec{M}$ was created for each time point separately as follows:

We first constructed a matrix $\varvec{V}$ based on the ASVs feature table with a dimension n × N, where n is the number of animals and N is the number of ASVs for each time point. Each element ${V}_{ij}$ represented relative abundance of ASV *j* in animal *i* for a given time point. A value of 0.01 was added to each ${V}_{ij}$ to guarantee positive definiteness, as described by Camarinha-Silva et al. [32]. Then, the matrix $\varvec{V}$ was used to calculate the elements of matrix $\varvec{X}$ with the same dimension as:

$${X}_{ij}= \frac{log{V}_{ij}- \stackrel{-}{log{V}_{.j}} }{sd\left(log{V}_{.j}\right)}$$

Thus, the matrix $\varvec{X}$ contained log-transformed, centered, and scaled relative abundance of ASV. The microbial relationship matrix **M** was then computed as $\varvec{M}= \frac{1}{N}\varvec{X}{\varvec{X}}^{\varvec{T}}$, representing the covariance between individuals based on their microbial composition resemblance.

We fitted the mixed models using a package BGLR [33] in R environment (v4.0.2) [30]. A Markov chain Monte Carlo (MCMC) algorithm was used to perform the analyses with 150,000 iterations, 50,000 iterations discarded as burn-in, and 10 iterations set as the thinning interval. Convergence of the models was visually checked by looking at trace plots of each parameter posterior distribution. The total phenotypic variance was obtained as the sum of σ${}_{s}^{2}$, ${{\upsigma }}_{p}^{2}$, ${{\upsigma }}_{m}^{2}$, and ${{\upsigma }}_{e}^{2}$. The proportion of phenotypic variance explained by the microbiota was calculated as the ${{\upsigma }}_{m}^{2}$ over the phenotypic variance.

$${m}^{2}= \frac{{{\upsigma }}_{m}^{2}}{{{\upsigma }}_{s}^{2}+{{\upsigma }}_{p}^{2}+{{\upsigma }}_{m}^{2}+{{\upsigma }}_{e}^{2} }$$

This proportion was defined with the term “microbiability” (m^2^) by Difford et al. [34, 35] and represented the overall interaction of the microbiota with the host phenotype. ANOVA was performed using the PROC GLM in SAS (v9.4, SAS Institute, Carry, NC, USA) to compare the microbiability estimates among feeding behavior traits, breeds, and time points, as well as their interactions. The least-squares means for fixed effects and contrasts were obtained with the LSMEANS statement of the PROC GLM. A P-value less than or equal to 0.05 was considered significant.

#### Association between ASVs and feeding behavior traits

To investigate the association between individual ASVs and feeding behavior traits, the following linear mixed model was constructed using the PROC GLIMMIX in SAS (v9.4, SAS Institute, Cary, NC, USA):

$${y}_{ijklmn}=\mu + {Breed}_{i}+{Room}_{j}+ {asv}_{k}+asv *{Breed}_{\left(ik\right)}+ {sire}_{l}+ {pen}_{m}+{e}_{ijklmn}$$

where ${y}_{ijklmn}$ is the feeding behavior trait at each time point, $\mu$ is the overall intercept, ${Breed}_{i}$ is the fixed effect of *i*^th^ breed, ${Room}_{j}$ is the fixed effect of *j*^th^ room, ${asv}_{k}$ is the covariate of the *k*^th^ ASV, $asv *{Breed}_{\left(ik\right)}$ is the fixed effect of the (*ik)*^th^ ASV and breed interaction, ${Sire}_{l}$ is the random effect of *l*^th^ sire within breed, ${Pen}_{m}$ is the random effect of *m*^th^ pen within room, and ${e}_{ijklmn}$ is the residual. The marginal effects of individual ASVs were evaluated, and the orthogonal contrasts were performed among breed groups at each time point. The Benjamini-Hochberg method was used to adjust the P-values to control the false discovery rate (FDR). An FDR of 0.05 was set as the cutoff threshold to declare the significance of the association between ASV and trait.

## Results

### Breed feeding behavior characteristics at three time points

**Fig. 1:**
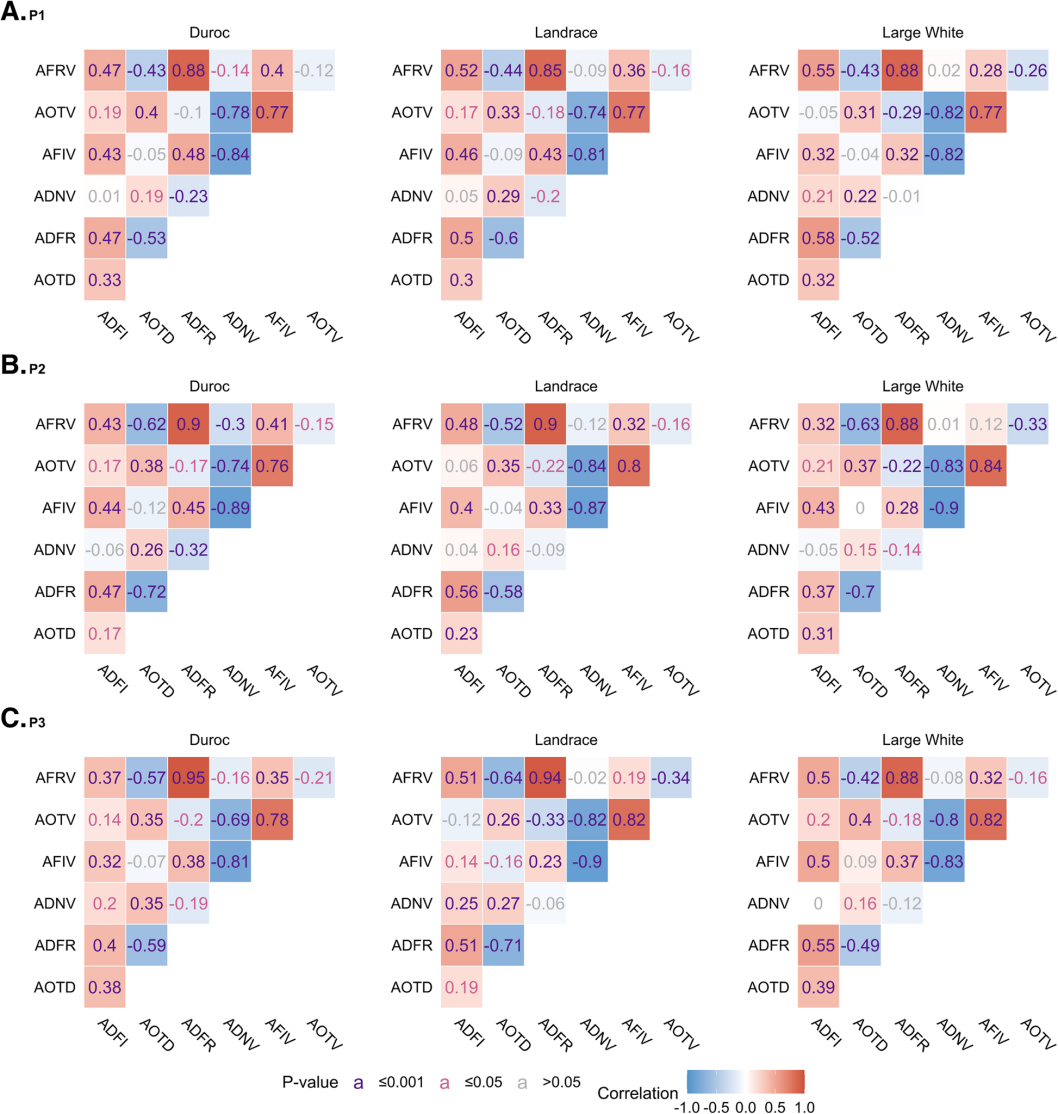
Spearman’s correlations of feeding behavior traits by breed during three periods. Correlations with P-value ≤ 0.001, ≤ 0.05, and > 0.05 are depicted in purple, pink, and grey, respectively. ADFI = average daily amount of feed consumed (g); AOTD = average daily feeder occupation time (s); ADFR = average daily feeding rate (g/min); ANVD = average daily number of visits to feeder; AFIV = average amount of feed consumed per visit (g); AOTV = average feeder occupation time per visit (s); AFRV = average feeding rate per visit (g/min). (**A**) Correlations of traits during P1; (**B**) Correlations of traits during P2; (**C**) Correlations of traits during P3

Phenotypic correlations for individual breed groups by period are depicted in Fig. 1. Generally, correlations between feeding behavior traits were similar across breeds during each period. Strong correlations were found between AFIV and AOTV (r > 0.75, P < 0.001), AFRV and ADFR (r > 0.85, P < 0.001), ANVD and AFIV (r < -0.80, P < 0.001), as well as ANVD and AOTV (r < - 0.70, P < 0.001). As expected, the feeding rate (ADFR and AFRV) had negative relationships with the feeder occupation time (AOTD and AOTV).

**Fig. 2:**
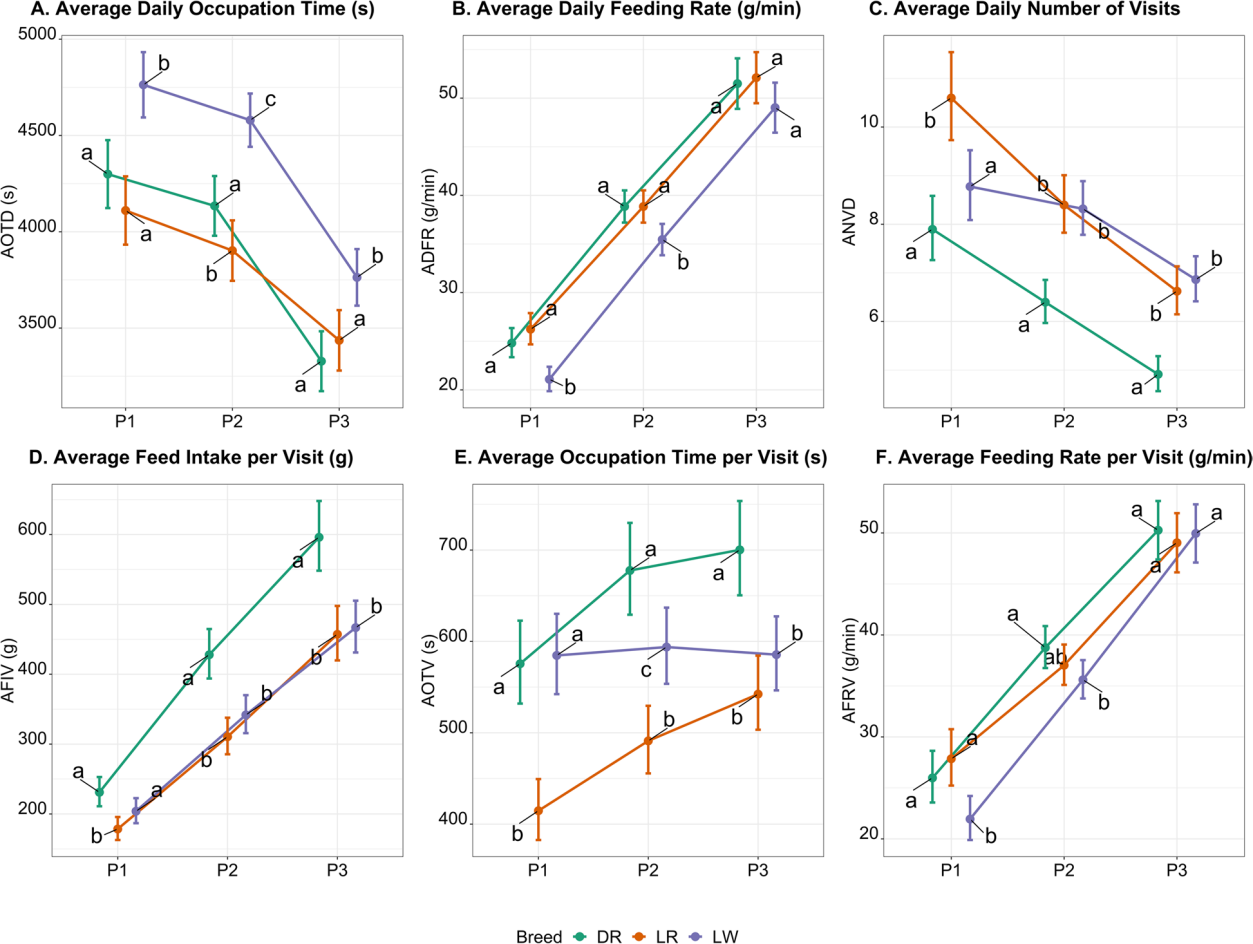
Contrasts of feeding behavior traits among breeds during three periods. Data are presented as least squares mean with the confidence interval. Different letters a, b, and c denote P < 0.05 between breeds within each time point. Colors represent three breeds: Duroc (DR), Landrace (LR), and Large White (LW). The x-axis represents three periods: P1, P2, and P3. To avoid the overlaps, dots representing three breeds are depicted horizontally away from each other for each period. (**A**) AOTD = average daily feeder occupation time (s); (**B**) ADFR = average daily feeding rate (g/min); (**C**) ANVD = average daily number of visits to feeder; (**D**) AFIV = average amount of feed consumed per visit (g); (**E**) AOTV = average feeder occupation time per visit (s); (**F**) AFRV = average feeding rate per visit (g/min)

The phenotypic differences of average daily feed intake (ADFI) among breeds have been reported in our previous study [23]. Thus, we excluded it and reported the results of the remaining six feeding behavior traits in the present study. Least squares means and 95% confidence intervals of feeding behavior traits for the effect of breed during each period are presented in Fig. 2. Significant differences between breeds were found for every feeding behavior trait during each period, except for feeding rate traits (ADFR and AFRV) during P3. The LW pigs spent more time in the feeder daily (AOTD) than the other two breeds across periods. The DR pigs visited the feeder less frequently (ANVD) but consumed more feed (AFIV) and spent more time visiting the feeder (AOTV) compared to the other breeds, especially during P2 and P3. The LW pigs had smaller feeding rates (ADFR and AFRV) than DR and LR pigs during P1 and P2, with no difference noticed during P3 among breeds. Differences in feeding behavior by room are depicted in Additional file 4. The ANOVA results of fixed effects breed and room are included in Additional file 5.

### Microbiability of host feeding behavior traits

**Fig. 3:**
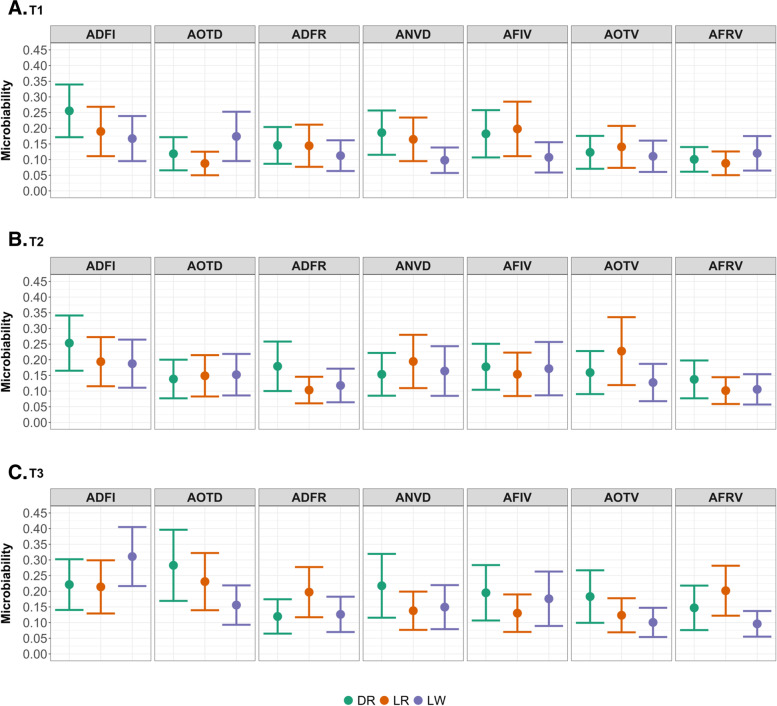
Estimated microbiability (m^2^) for feeding behavior traits by breed and time points. Data are presented as mean with SE error bars. Colors represent three breeds: Duroc (DR), Landrace (LR), and Large White (LW). ADFI = average daily amount of feed consumed (g); AOTD = average feeder daily occupation time (s); ADFR = average daily feeding rate (g/min); ANVD = average daily number of visits to feeder; AFIV = average amount of feed consumed per visit (g); AOTV = average feeder occupation time per visit (s); AFRV = average feeding rate per visit (g/min). (**A**) The microbiability values at 73 days (T1); (**B**) The microbiability values at 123 days (T2); (**C**) The microbiability values at 158 days (T3)

Relative abundance of the gut microbial taxa of DR, LR, and LW pigs at T1, T2 and T3 is described in our previous study [23]. The relative proportion of the phenotypic variation in host feeding behavior traits attributed to gut microbial composition, defined as “microbiability” (m^2^), is depicted by breed and time point in Fig. 3. The m^2^ estimates ranged from 0.10 (SE = 0.04) to 0.28 (SE = 0.11) for DR pigs, 0.09 (SE = 0.04) to 0.23 (SE = 0.09) for LR pigs, and 0.10 (SE = 0.04) to 0.31 (SE = 0.09) for LW pigs across the three time points. Results of differences in m^2^ estimates across breeds, sampling time points, or feeding behavior traits are summarized in Additional file 6. The m^2^ estimates were similar between DR and LR (P = 0.42), as well as between LR and LW (P = 0.32), but they were greater for DR than LW (P = 0.02). When the m^2^ estimates were compared across the three time points, they were similar between T1 and T2 (P = 0.34) and between T2 and T3 (P = 0.28), while they were significantly higher at T3 than T1 (P = 0.01). Furthermore, compared to other feeding behavior traits, the gut microbial composition explained a greater proportion of the total variation in ADFI. There were no significant interaction effects between breed, time point, and feeding behavior trait. Estimates of variance components for each trait obtained from two models are depicted in Additional file 7. Compared to the baseline model, the inclusion of microbial information in the model helped reduce the proportion of variance previously included in the residual component for most feeding behavior traits.

### Specific microbes associated with feeding behavior traits

**Fig. 4:**
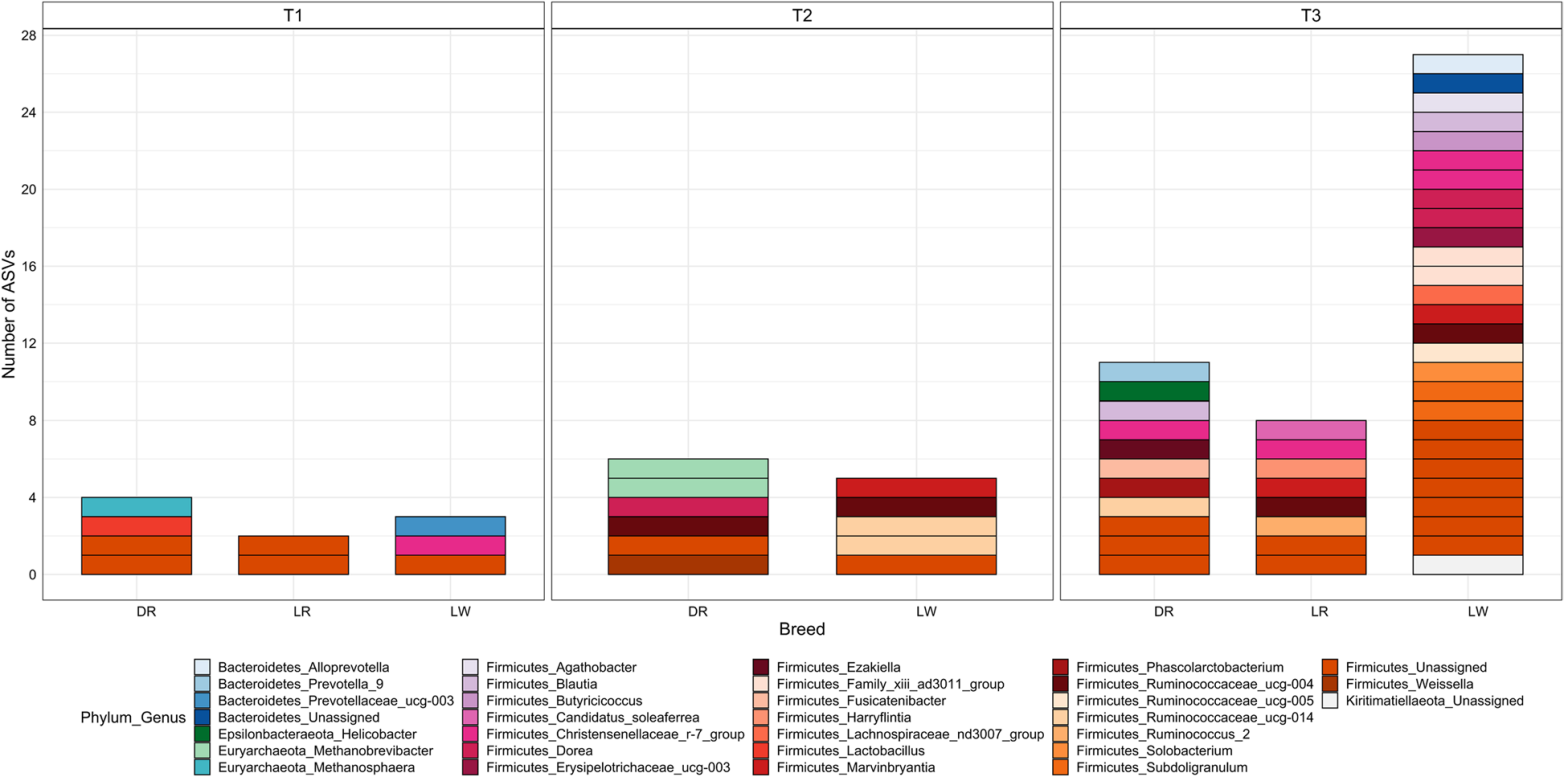
Taxonomy of ASVs significantly associated with feeding behavior traits by breed at each time point. Taxonomy is depicted at the combination of genus and phylum level. The x-axis represents three breeds: Duroc (DR), Landrace (LR), and Large White (LW). The y-axis represents the number of identified ASVs for each category. Colors from the same color scheme represent different genus levels from the same phylum

A false discovery rate of 0.05 was considered the threshold to identify ASVs significantly associated with feeding behavior traits. The relationship between the gut microbiota and ADFI was reported in our previous study [23]. Thus, we only reported the results of the other feeding behavior traits in this section. Within each breed group, we identified 4, 6, and 11 ASVs for DR pigs, 2, 0, and 8 ASVs for LR pigs, and 3, 5, and 27 ASVs for LW pigs at T1, T2, and T3 associated with feeding behavior traits, respectively (Table 1). The majority of the identified ASVs were associated with AOTD for all breed groups at T3. However, at the threshold of FDR 0.05 or 0.10, no ASVs were identified associated with ADFR in all the breed groups across the three time points. The taxonomy of identified ASVs is depicted in Fig. 4 in terms of the combination of phylum and genus annotations by breed group. At the phylum level, the identified ASVs were annotated to *Bacteroidetes, Epsilonbacteraeota, Euryarchaeota*, and *Firmicutes* for DR pigs, *Firmicutes* for LR pigs, *Bacteroidetes, Firmicutes*, and *Kiritimatiellaeota* for LW pigs across the three time points. Most of the ASVs were assigned to *Firmicutes*, then *Bacteroidetes, Euryarchaeota*, *Kiritimatiellaeota*, and *Euryarchaeota*. Except for a small number of identified ASVs that were not assigned to known genus, the rest of them were annotated to 14, 6, and 16 genus levels for DR, LR, and LW pigs across the three time points, respectively.

**Table 1:** Number of ASVs significantly associated with feeding behavior traits by breed and sampling time point

Trait^a^	Time Point	*p*-value < .05			*q*-value^b^ < .05		
		DR^c^	LR	LW	DR	LR	LW
AOTD (s)	T1	59	30	57	2	1	0
	T2	104	101	112	4	0	5
	T3	105	131	221	6	8	26
ADFR (g/min)	T1	81	39	33	0	0	0
	T2	80	30	26	0	0	0
	T3	58	79	46	0	0	0
ANVD	T1	59	43	23	0	0	0
	T2	46	62	35	0	0	0
	T3	82	54	89	1	0	1
AFIV (g)	T1	51	52	24	0	1	0
	T2	88	34	60	1	0	0
	T3	66	27	69	1	0	0
AOTV (s)	T1	29	38	36	0	0	0
	T2	61	64	57	0	0	0
	T3	56	52	44	1	0	0
AFRV (g/min)	T1	52	25	54	2	0	3
	T2	87	23	30	1	0	0
	T3	55	74	26	2	0	0
Total	T1	331	227	227	**4**	**2**	**3**
	T2	466	314	320	**6**	**0**	**5**
	T3	422	417	495	**11**	**8**	**27**

^a^ *AOTD* average daily feeder occupation time (s); *ADFR *average daily feeding rate (g/min); *ANVD *average daily number of visits to feeder; *AFIV *average amount of feed consumed (g) per visit; *AOTV *average feeder occupation time (s) per visit; *AFRV *average feeding rate (g/min) per visit. ^b^ FDR value = *p*-value adjusted by the Benjamini-Hochberg method. ^c^ *DR* Duroc; *LR* Landrace; *LW* Large White

**Fig. 5:**
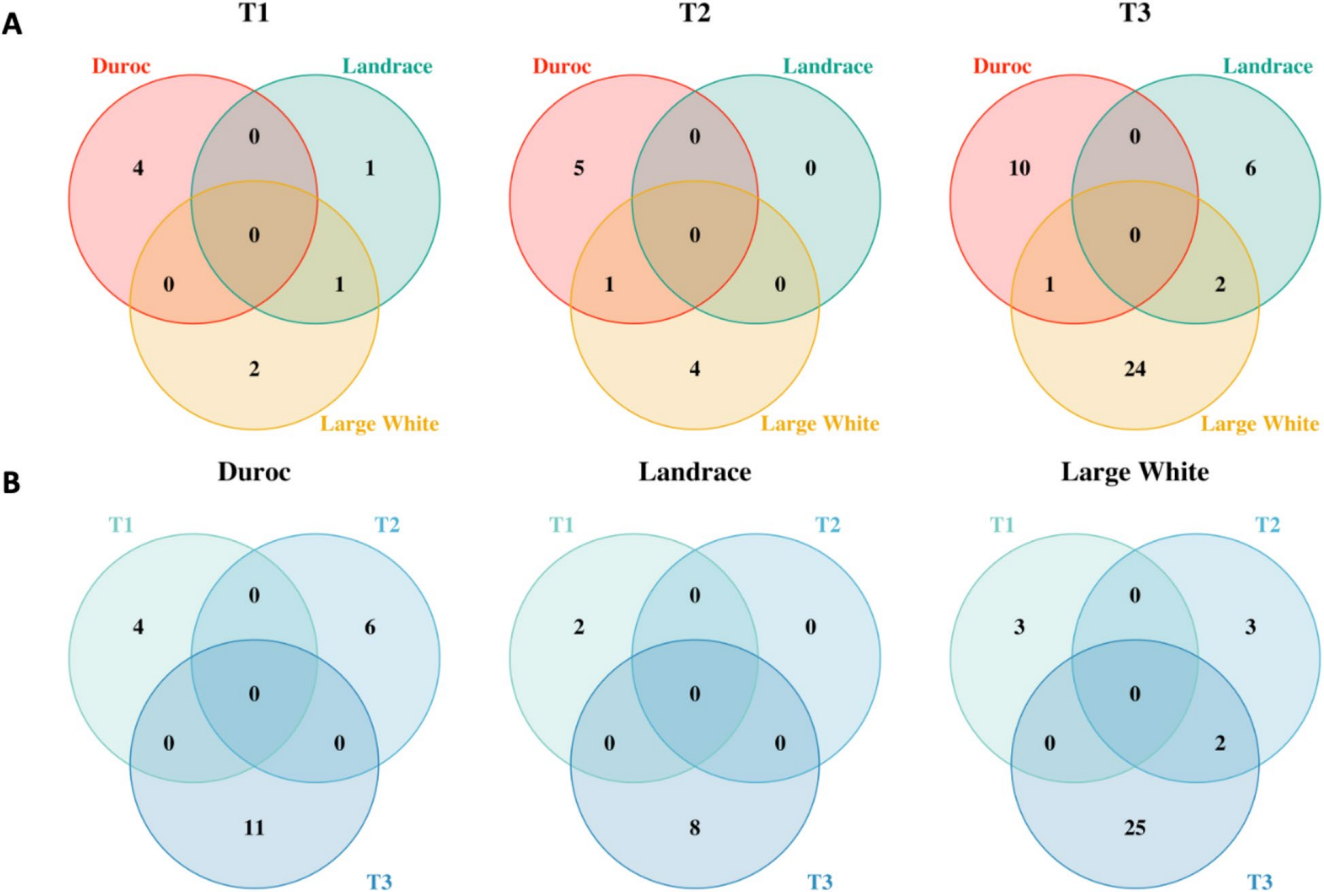
Venn diagram of common and unique ASVs significantly associated with feeding behavior traits. (**A**) Across breed groups at each time point; (**B**) Across time points within each breed group

Venn diagrams of the number of common and unique identified ASVs among breed groups and time points are presented in Fig. 5. Identified ASVs were breed-specific at each time point with a minor overlapping among breed groups. There was only one ASV in common between LR and LW pigs at T1, and one ASV was shared between DR and LW pigs at T2 (Fig. 5 A). Three ASVs were shared by LW pigs with either DR or LR pigs, and no overlapping was found between DR and LR pigs at T3(Figure 5 A). Similarly, when comparing identified ASVs within the breed group across time points, only two ASVs were found the same between T2 and T3 in LW pigs, and no shared ASV was found across time points in DR and LR pigs (Fig. 5B).

**Fig. 6:**
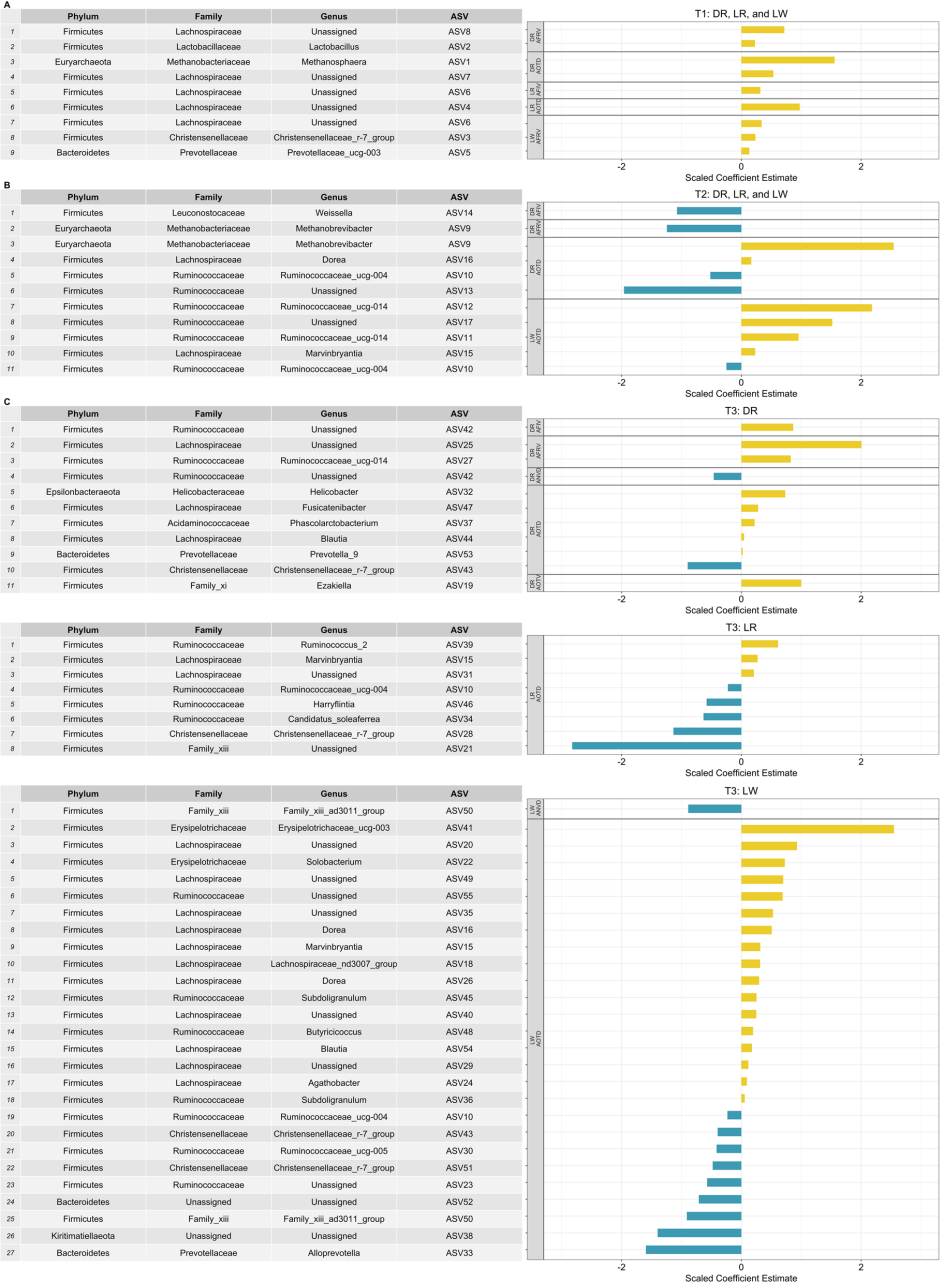
The coefficient estimates of identified ASVs (FDR < 0.05) by breed at three time points. Scaled data are presented to make a comparison on a similar scale. Each ASV is referred by taxonomy (phylum, family, genus) and ASV number in the table corresponding to the plot on the right. (**A**) Combined results for Duroc (DR), Landrace (LR), and Large White (LW) at 73 days (T1); (**B**) Combined results for Duroc (DR), Landrace (LR), and Large White (LW) at 123 days (T2); (**C**) Results for Duroc (DR), Landrace (LR), and Large White (LW) at 158 days (T3)

The scaled coefficient estimates of identified ASVs by breed group at three time points are depicted in Fig. 6. A total of 55 unique ASVs were either positively or negatively associated with feeding behavior traits. Nine ASVs were positively associated with AFRV (n = 5), AOTD (n = 3), and AFIV (n = 1) in three breed groups at T1, and most of them were annotated to *Lachnospiraceae* family belonging to *Firmicutes* phylum (Fig. 6 A). Five ASVs negatively associated with AFIV (n = 1), AFRV (n = 1), and AOTD (n = 2) for DR pigs and AOTD (n = 1) for LW pigs at T2 were annotated to *Methanobrevibacter, Weissella, and Ruminococcaceae*_UCG-004 at the genus level. Besides, six ASVs positively associated with AOTD for DR and LW pigs were annotated to *Methanobrevibacter* (*Euryarchaeota* phylum), *Dorea, Marvinbryantia*, and *Ruminococcaceae* (*Firmicutes* phylum) at the genus level (Fig. 6B). At T3, 29 ASVs with positive associations across breeds were annotated to 16 genus levels and mainly classified to either *Blautia, Dorea, Ruminococcus*_2, and *Subdol*. Most of the 17 ASVs with negative associations in three breeds were annotated to *Christensenellaceae*_R-7_group, *Family*_XIII_AD3011_group, and *Ruminococcaceae*_UCG-004 genus in *Firmicutes* phylum.

Among the 55 ASVs, eight were associated with feeding behavior traits across several breeds or time points (Fig. 6). For example, the ASV10 assigned to *Ruminococcaceae*_UCG-004 genus was adversely associated with AOTD in DR and LR at T2 and in LR and LW at T3. Similarly, the ASV43 assigned to *Christensenellaceae*_R-7_group was negatively associated with AOTD in DR and LW at T3. Additionally, the ASV15 belonging to *Marvinbryantia* genus and the ASV16 belonging to *Dorea* genus were positively associated with AOTD in LR and LW at T3, and in DR at T2 and LW at T3, respectively. Furthermore, several ASVs were associated with more than one feeding behavior trait; ASV6 (unassigned genus) with AFIV in LR and AFRV in LW at T1; ASV9 (*Methanobrevibacter* genus) with AFRV and AOTD in DR at T2; ASV42 (unassigned genus) with ANVD and AFIV in DR at T3; and ASV50 (*Family*_XIII_AD3011_group genus) with ANVD and AOTD in LW at T3. The contrasts of the coefficient estimate for the most significant ASV (least FDR) in each trait-breed-time point category are depicted in Additional file 8.

## Discussion

Phenotypic correlations between feeding behavior traits in this study are similar to the results previously reported by Fernández et al. [14]. In agreement with Rauw et al. [36] and Carcò et al. [37], we observed an increase in both the feeding rate and feed consumption of pigs with age, most likely due to their increased physical capacity. As expected, we observed different feeding patterns between breeds. We found that Large White pigs had a lower feeding rate than Duroc and Landrace pigs during the first two periods. In contrast, Fernández et al. [14] found that Duroc had lowest feeding rate of the three breeds, defining them as “slow eaters”. Variations in feeding behaviors among rooms or pens for growing pigs were noticed in this study. It is expected that with only one Feeder equipped per pen, social ranks in group-housed pigs may lead to competition at feeding and result in a discrepancy in feeding behavior across pens [38].

First proposed by Difford et al. [34], estimation of microbiability has recently become a popular approach to quantify the proportion of variation in host phenotypes explained by the microbial composition in agricultural animal species. Difford et al. [34] reported that the ruminal microbiota composition was responsible for 15% of the total phenotypic variation in CH_4_ emissions of dairy cows. Wen et al. [39] indicated that the duodenal and cecal microbiota explained 24% and 21% of fat deposition variation in chicken, respectively. Moreover, Vollmar et at. [40] reported that the variations in the gut microbiota were associated with 9%, 18%, and 27% of variations in the feed intake, daily gain, feed per gain ratio in Japanese Quail, respectively. Our microbiability estimates ranged from 0.09 to 0.31, which suggested that the gut microbiota was associated with a small to moderate amount of variation in host feeding behavior among animals. In this study, breed effects on microbiability were discovered. Differences in the microbiability estimates between Duroc and Large White pigs suggest that the gut microbial composition may differ in predicting the host phenotype in populations with diverse genetic backgrounds. Furthermore, our findings show that the gut microbial composition of pigs at the finishing stage (T3) is more informative in explaining the variations in feeding behavior than at the start of the growth period (T1). The differences in the microbiability estimates across time points could be explained by several factors. For example, if all microbial samples had been collected at a uniform time point (i.e., the midpoint) within each feeding behavior measurement period, we might have seen fewer variances in the microbiability. In addition, transitions in the diet and environment prior to our first sampling may have contributed to differences in the microbiability between growth stages. We also observed variations in the microbiability estimates across feeding behavior traits, with the highest values for average daily feed intake, ranging from 0.17 to 0.31, which are similar to the estimate of 0.16 for feed intake of Pietrain sows at the slaughter age estimated by Camarinha-Silva et al. [32]. To the best of our knowledge, our study is the first one to estimate the microbiability for feeder occupation time, feeding rate, and number of visits to the feeder in multiple breeds at three time points during the growth in swine. These findings suggest that host genetics and age may have an impact on microbiability for different traits in pigs, but more research is needed to confirm the findings in different populations.

Previous studies have suggested that the gut microbial composition varies among individuals due to breed and age in pigs [15, 16, 23]. In this study, the identified ASVs associated with feeding behavior were also breed-specific and stage-specific during the growing to finishing period, with a small part of ASVs shared among breeds or sampling time points. At the last sampling time point (158 days of age), the greatest number of ASVs were found associated with feeding behavior across breeds compared to the previous two sampling time points (73 and 123 days of age). This might be due to the relative higher alpha diversity measured by the Shannon Index and greater number of clusters in the gut microbiota at 158 days compared to early time points in all three breeds, as described in our previous study [23].

Among the 40 ASVs positively associated with feeding behavior, more than half of the ones associated with feed intake, feeding rate, and feeder occupation time were assigned to *Lachnospiraceae* family in *Firmicutes* phylum. Similarly, Cox et al. [41] identified four ASVs in *Lachnospiraceae* family that exhibited greater abundances in people with good appetite than people with poor appetite by comparing their gut microbial composition. At the genus level, two ASVs robust in positive associations with the daily feeder occupation time in multiple breeds and time points were annotated to *Marvinbryantia* and *Dorea*. The remaining identified ASVs positively associated with the daily feeder occupation time were mainly annotated to *Blautia* and *Ruminococcaceae*_UCG-014. Interestingly, these microbes have been found correlated to complex host behaviors, such as stressor-induced behavior and depressive-like behavior in human and animal models [42–45]. Shifts in the abundance of *Blautia* [43], *Dorea* [42], and *Marvinbryantia* [45] were observed in stressed and depressed mice. Similarly, reduction in the abundance of *Ruminococcaceae*_UCG-014 was linked to the development of anxiety-like behaviors in humans [44]. The genus *Prevotella* positively associated with the daily feeder occupation time of Duroc pigs in our study were also found positively correlated with the appetite of Duroc pigs in a previous study conducted by Yang et al. [46]. One ASV belonging to *Lactobacillus* genus was found to associate with the daily feeding rate in Duroc pigs at 73 days of age in this study. *Lactobacillus* strains, commonly used as probiotics promoting overall intestinal health, have been found to influence the host eating behavior through various pathways [47, 48].

The present study also identified 17 ASVs that had negative associations with host feeding behavior traits. At the family level, the majority of these ASVs were classified into *Ruminococcaceae, Christensenellaceae*, or *Family*_XIII. Numerous studies have indicated that the intestinal abundance of *Christensenellaceae* is negatively related to host body mass index and fat mass in human studies [49–51]. Similarly, a significant number of microbes belonging to *Ruminococcaceae* family colonized in the gut have been associated with the lower weight gain and lean phenotype in humans [52]. In our study, several microbes from the *Ruminococcaceae* and *Christensenellaceae* families were negatively associated with daily feeder occupation time or the number of visits to the feeder. It is possible that the reduced feeding behavior might be linked to the low fatness deposit and weight gain through specific gut taxa, but further studies are needed in this sense. Moreover, both positive and negative associations were identified between microbes from *Ruminococcaceae* family and feeding behavior measures, including daily feeder occupation time, feed intake per visit to the feeder, and feeding rate across breeds and time points in this study. Similarly, Yang et al. [46] reported one positive and two negative associations between *Ruminococcaceae* family members and feed intake in pigs. These findings suggest that *Ruminococcaceae* family may contain a diverse range of members in the gut, each with distinct functions. Further investigations on the specific individuals belonging to *Ruminococcaceae* family are needed to clarify this aspect. At the genus level, three ASVs involved in multiple negative associations with the daily feeder occupation time and number of visits to the feeder in several breeds and time points were annotated to *Ruminococcaceae*_UCG-004, *Christensenellaceae*_R-7_group, and *Family*_XIII_AD3011_group. Among these microbes, *Christensenellaceae*_R-7_group [53], *Ruminococcaceae*_UCG-004 [54] are known to produce short-chain fatty acids, which are essential metabolites in regulating host energy homeostasis and appetite through gut-brain communication in the gut [55]. Accumulating research has indicated that elevated SCFAs production in the gut could suppress the host appetite [55–57], thus modifying feeding behavior. We identified an ASV belonging to *Methanobrevibacter* genus in a negative association with the average feeding rate per visit and also in a positive association with the average daily feeder occupation time in Duroc pigs at 123 days in the present study. Interestingly, several studies have shown that people with anorexia nervosa have higher levels of *Methanobrevibacter* in their gut [58, 59]. The microbes found in this study could serve as candidates for further clarifications on the relationship between the gut microbiota and host feeding behavior. However, differences in the environment [60], experimental design [61], the choice of sequencing platform [62], and 16 S target regions [63] limit the comparisons of results from various microbiome studies. Thus, more studies are still needed to validate the results in different populations with various conditions and further establish the causality between specific microbes and feeding behavior in swine.

## Conclusions

In this study, we observed different feeding patterns recorded by the electronic feeder among Duroc, Landrace, and Large White pigs at three time points during the growing-finishing period. By modeling the microbial relationship among animal individuals and estimating the microbiability, we quantified a low to moderate amount of variation in host feeding behavior associated with the gut microbial composition in each breed group across the three time points. Moreover, we identified a total of 55 unique microbes differently associated with feed intake, feeder occupation time, number of visits to the feeder, and feeding rate in three breed groups, which might provide information for future studies aiming at modulating feeding disorders and improving production in growing pigs. Our study provides insights to better understand the importance of the gut microbiota in interacting with host feeding behavior and highlights the influences of host genetic background and age in porcine microbiome studies.

## Supplementary Information

**Additional file 1.** The family relationship among pigs for each breed based on pedigree. The numerator relationship matrices were calculated from pedigree information and is presented in individual sheet for each breed.

**Additional file 2.** Distribution of animals for three breeds kept in each room. The y-axis represents the number of animals from each breed group kept in each room. The x-axis represents the room (n=8). Colors represent three breeds: Duroc (green), Landrace (orange), and Large White (purple).

**Additional file 3.** Ingredient composition for the standard diet for pigs during the present study period. The diet ingredients listed in the table were used to feed all the animals in the present study throughout the experimental period (73 to 163 days of age). This diet was provided as of 70 days of age until pigs reached the final body weight (127 kg).

**Additional file 4.** Contrasts of estimates for feeding behavior traits among rooms during three time periods.Data are presented as least squares mean with the confidence interval. Colors represent different rooms (n = 8).

**Additional file 5.** Summary of F values and P-values from ANOVA of fixed effects on feeding behavior traits. F values are calculated as the Mean Square Model divided by the Mean Square Residual in the ANOVA test from SAS. ADFI = average daily amount of feed consumed (g); AOTD = average daily feeder occupation time (s); ADFR = average daily feeding rate (g/min); ANVD = average daily number of visits to feeder; AFIV = average amount of feed consumed (g) per visit; AOTV = average feeder occupation time (s) per visit; AFRV = average feeding rate (g/min) per visit. * P <.05; **P < .01; ***P < .001.

**Additional file 6.** Comparisons of least squares means of microbiability across breeds, time points, andfeeding behavior traits. DR = Duroc (n = 205); LR = Landrace (n = 226); LW =Large White (n = 220). T1 = 73 days of age; T2 = 123 days of age; T3 = 158 daysof age. ADFI = average amount of feed consumed (g) daily; AOTD = average daily feeder occupation time (s); ADFR = average daily feeding rate (g/min); ANVD = average daily number of visits to feeder; AFIV = average amount of feed consumed (g) per visit; AOTV = average feeder occupation time (s) per visit; AFRV = average feeding rate (g/min) per visit. Different letters a, b, and c denote P <0.05 between means in the row.

**Additional file 7.** Proportions of variance component estimates for feeding behavior traits at three timepoints. The plot depicts the proportions of total phenotypic variance explained by pen and sire in Null model and pen, sire, and gut microbiota in MBLUP model for Duroc (DR), Landrace (LR), and Large White (LW) at 73 days (T1), 123 days (T2),and 158 days (T3). P = the proportion of variance explained by pen effect; S =the proportion of variance explained by sire effect; M = the proportion of variance explained by the gut microbial composition; R = residual.

**Additional file 8.** Coefficients of identified ASVs significantly associated with feeding behavior traits at three time points. The plot depicts coefficients of the most significant ASV (least FDR) for each feeding behavior trait by breed at 73 days (T1), 123 days(T2), and 158 days (T3). Data are presented as estimated coefficients with a 95% confidence interval. Each ASV is represented using the combination of phylum, genus, and ASV number.

## Data Availability

The phenotypic datasets that support the finding of this study are available from Smithfield Premium Genetics (Rose Hill, NC, USA). Restrictions apply to the availability of these data, which were used under license for the current study, and so are not publicly available. Data are, however, available from the authors upon reasonable request and with permission of Smithfield Premium Genetics. A Smithfield Premium Genetics request for accessing data may be sent to Kent Gray, General Manager (kgray@smithfield.com). The 16 S rRNA gene sequencing datasets generated and analyzed during the current study are available in the NCBI repository, with the accession number PRJNA747026.

## Abbreviations

ASV: Amplicon sequence variant
DR: Duroc
LR: Landrace
LW: Large White
ADFI: Average daily feed intake
AOTD: Average daily feeder occupation time
ADFR: Average daily feeding rate
ANVD: Average daily number of visits to the feeder
AFIV: Average feed intake per visit
AOTV: Average feeder occupation time per visit
AFRV: Average feeding rate per visit

## References

[1] Fetissov SO (2017). Role of the gut microbiota in host appetite control: bacterial growth to animal feeding behaviour. Nat Rev Endocrinol..

[2] Brown-Brandl TM, Rohrer GA, Eigenberg RA (2013). Analysis of feeding behavior of group housed growing-finishing pigs. Comput Electron Agric.

[3] Hyun Y, Ellis M (2002). Effect of group size and feeder type on growth performance and feeding patterns in finishing pigs. J Anim Sci.

[4] Carcò G, Gallo L, Dalla Bona M, Latorre MA, Fondevila M, Schiavon S (2018). The influence of feeding behaviour on growth performance, carcass and meat characteristics of growing pigs. PLoS One.

[5] Do DN, Strathe AB, Jensen J, Mark T, Kadarmideen HN (2013). Genetic parameters for different measures of feed efficiency and related traits in boars of three pig breeds. J Anim Sci.

[6] Lu D, Jiao S, Tiezzi F, Knauer M, Huang Y, Gray KA (2017). The relationship between different measures of feed efficiency and feeding behavior traits in Duroc pigs1. J Anim Sci.

[7] Miroschnikow A, Schlegel P, Pankratz MJ (2020). Making Feeding Decisions in the Drosophila Nervous System. Current Biology.

[8] Goulet O (2015). Potential role of the intestinal microbiota in programming health and disease. Nutr Rev.

[9] Patil Y, Gooneratne R, Ju XH (2020). Interactions between host and gut microbiota in domestic pigs: a review. Gut Microbes.

[10] van de Wouw M, Schellekens H, Dinan TG, Cryan JF (2017). Microbiota-gut-brain axis: Modulator of host metabolism and appetite. Journal of Nutrition.

[11] Kim JS, de La Serre CB (2018). Diet, gut microbiota composition and feeding behavior. Physiol Behav.

[12] David LA, Maurice CF, Carmody RN, Gootenberg DB, Button JE, Wolfe BE (2014). Diet rapidly and reproducibly alters the human gut microbiome. Nature.

[13] Zarrinpar A, Chaix A, Yooseph S, Panda S (2014). Diet and feeding pattern affect the diurnal dynamics of the gut microbiome. Cell Metab.

[14] Fernández J, Fàbrega E, Soler J, Tibau J, Ruiz JL, Puigvert X (2011). Feeding strategy in group-housed growing pigs of four different breeds. Appl Anim Behav Sci.

[15] Xiao Y, Li K, Xiang Y, Zhou W, Gui G, Yang H (2017). The fecal microbiota composition of boar Duroc, Yorkshire, Landrace and Hampshire pigs. Asian-Australasian J Anim Sci.

[16] Li Y, Wang X, Wang X, Wang J, Zhao J (2020). Life-long dynamics of the swine gut microbiome and their implications in probiotics development and food safety. Gut Microbes.

[17] Wang X, Tsai T, Deng F, Wei X, Chai J, Knapp J (2019). Longitudinal investigation of the swine gut microbiome from birth to market reveals stage and growth performance associated bacteria. Microbiome.

[18] Zhao W, Wang Y, Liu S, Huang J, Zhai Z, He C (2015). The dynamic distribution of porcine microbiota across different ages and gastrointestinal tract segments. PLoS One.

[19] Kim HB, Isaacson RE (2015). The pig gut microbial diversity: Understanding the pig gut microbial ecology through the next generation high throughput sequencing. Veterinary Microbiology.

[20] Pajarillo EAB, Chae JP, Balolong MP, Kim HB, Kang DK (2011). Assessment of fecal bacterial diversity among healthy piglets during the weaning transition. J Gen Appl Microbiol..

[21] Petri D, Hill JE, Van Kessel AG (2010). Microbial succession in the gastrointestinal tract (GIT) of the preweaned pig. Livest Sci.

[22] Lu D, Tiezzi F, Schillebeeckx C, McNulty NP, Schwab C, Shull C (2018). Host contributes to longitudinal diversity of fecal microbiota in swine selected for lean growth. Microbiome.

[23] Bergamaschi M, Tiezzi F, Howard J, Huang YJ, Gray KA, Schillebeeckx C (2020). Gut microbiome composition differences among breeds impact feed efficiency in swine. Microbiome.

[24] Faith JJ, Guruge JL, Charbonneau M, Subramanian S, Seedorf H, Goodman AL, et al. The Long-Term Stability of the Human Gut Microbiota. Science (80-). 2013;341:1237439. doi:10.1126/science.1237439. 10.1126/science.1237439 PMC3791589 23828941

[25] Casey DS, Stern HS, Dekkers JCM (2005). Identification of errors and factors associated with errors in data from electronic swine feeders. J Anim Sci.

[26] Magoc T, Salzberg SL (2011). FLASH: fast length adjustment of short reads to improve genome assemblies. Bioinformatics.

[27] Caporaso JG, Kuczynski J, Stombaugh J, Bittinger K, Bushman FD, Costello EK (2010). QIIME allows analysis of high-throughput community sequencing data. Nat Methods.

[28] Callahan BJ, McMurdie PJ, Rosen MJ, Han AW, Johnson AJA, Holmes SP (2016). DADA2: high-resolution sample inference from Illumina amplicon data. Nat Methods.

[29] Quast C, Pruesse E, Yilmaz P, Gerken J, Schweer T, Yarza P (2013). The SILVA ribosomal RNA gene database project: Improved data processing and web-based tools. Nucleic Acids Res.

[30] Team RC. R: A language and environment for statistical computing. 2021.

[31] Artusi R, Verderio P, Marubini E (2002). Bravais-Pearson and Spearman correlation coefficients: Meaning, test of hypothesis and confidence interval. International Journal of Biological Markers.

[32] Camarinha-Silva A, Maushammer M, Wellmann R, Vital M, Preuss S, Bennewitz J (2017). Host genome influence on gut microbial composition and microbial prediction of complex traits in pigs. Genetics.

[33] Pérez P, De Los Campos G (2014). Genome-wide regression and prediction with the BGLR statistical package. Genetics.

[34] Difford GF, Plichta DR, Løvendahl P, Lassen J, Noel SJ, Højberg O (2018). Host genetics and the rumen microbiome jointly associate with methane emissions in dairy cows. PLOS Genet.

[35] Difford G, Lassen J, Løvendahl P. Genes and microbes, the next step in dairy cattle breeding. In: Book of Abstracts of the 67th Annual Meeting of the European Federation of Animal Science. 2016. p. 285.

[36] Rauw WM, Soler J, Tibau J, Reixach J, Gomez Raya L (2006). Feeding time and feeding rate and its relationship with feed intake, feed efficiency, growth rate, and rate of fat deposition in growing Duroc barrows1. J Anim Sci.

[37] Carcò G, Gallo L, Bona MD, Latorre MA, Fondevila M, Schiavon S. The influence of feeding behaviour on growth performance, carcass and meat characteristics of growing pigs. PLoS One. 2018;13. doi:10.1371/journal.pone.0205572. 10.1371/journal.pone.0205572 PMC6188860 30321211

[38] Georgsson L, Svendsen J (2002). Degree of competition at feeding differentially affects behavior and performance of group-housed growing-finishing pigs of different relative weights. J Anim Sci.

[39] Wen C, Yan W, Sun C, Ji C, Zhou Q, Zhang D (2019). The gut microbiota is largely independent of host genetics in regulating fat deposition in chickens. ISME J.

[40] Vollmar S, Wellmann R, Borda-Molina D, Rodehutscord M, Camarinha-Silva A, Bennewitz J. The gut microbial architecture of efficiency traits in the domestic poultry model species Japanese quail (coturnix japonica) assessed by mixed linear models. G3 Genes, Genomes, Genet. 2020;10:2553–62. doi:10.1534/g3.120.401424. 10.1534/g3.120.401424 PMC7341145 32471941

[41] Cox NJ, Bowyer RCE, Ni Lochlainn M, Wells PM, Roberts HC, Steves CJ. The composition of the gut microbiome differs among community dwelling older people with good and poor appetite. J Cachexia Sarcopenia Muscle. 2021;:jcsm.12683. doi:10.1002/jcsm.12683. 10.1002/jcsm.12683 PMC8061352 33580637

[42] McGaughey KD, Yilmaz-Swenson T, Elsayed NM, Cruz DA, Rodriguiz RM, Kritzer MD (2019). Relative abundance of Akkermansia spp. and other bacterial phylotypes correlates with anxiety-and depressive-like behavior following social defeat in mice. Sci Rep.

[43] Wong ML, Inserra A, Lewis MD, Mastronardi CA, Leong L, Choo J (2016). Inflammasome signaling affects anxiety- and depressive-like behavior and gut microbiome composition. Mol Psychiatry.

[44] Chen Y huan, Bai J, Wu D, Yu S fen, Qiang X ling, Bai H, et al. Association between fecal microbiota and generalized anxiety disorder: Severity and early treatment response. J Affect Disord. 2019;259:56–66. 10.1016/j.jad.2019.08.014 31437702

[45] Yu M, Jia H, Zhou C, Yang Y, Zhao Y, Yang M (2017). Variations in gut microbiota and fecal metabolic phenotype associated with depression by 16S rRNA gene sequencing and LC/MS-based metabolomics. J Pharm Biomed Anal.

[46] Yang H, Yang M, Fang S, Huang X, He M, Ke S (2018). Evaluating the profound effect of gut microbiome on host appetite in pigs. BMC Microbiol.

[47] Alcock J, Maley CC, Aktipis CA (2014). Is eating behavior manipulated by the gastrointestinal microbiota? Evolutionary pressures and potential mechanisms. BioEssays.

[48] Narmaki E, Borazjani M, Ataie-Jafari A, Hariri N, Doost AH, Qorbani M (2020). The combined effects of probiotics and restricted calorie diet on the anthropometric indices, eating behavior, and hormone levels of obese women with food addiction: a randomized clinical trial. Nutr Neurosci.

[49] Fu J, Bonder MJ, Cenit MC, Tigchelaar EF, Maatman A, Dekens JAM (2015). The gut microbiome contributes to a substantial proportion of the variation in blood lipids. Circ Res.

[50] Le Roy CI, Beaumont M, Jackson MA, Steves CJ, Spector TD, Bell JT (2018). Heritable components of the human fecal microbiome are associated with visceral fat. Gut Microbes.

[51] Hibberd AA, Yde CC, Ziegler ML, Honoré AH, Saarinen MT, Lahtinen S (2019). Probiotic or synbiotic alters the gut microbiota and metabolism in a randomised controlled trial of weight management in overweight adults. Benef Microbes.

[52] Menni C, Jackson MA, Pallister T, Steves CJ, Spector TD, Valdes AM (2017). Gut microbiome diversity and high-fibre intake are related to lower long-term weight gain. Int J Obes.

[53] Zhang X, Wu Y, Ye H, Feng C, Han D, Tao S (2020). Dietary milk fat globule membrane supplementation during late gestation increased the growth of neonatal piglets by improving their plasma parameters, intestinal barriers, and fecal microbiota. RSC Adv.

[54] Han H, Zhou Y, Liu Q, Wang G, Feng J, Zhang M (2021). Effects of Ammonia on Gut Microbiota and Growth Performance of Broiler Chickens. Animals.

[55] Byrne CS, Chambers ES, Morrison DJ, Frost G (2015). The role of short chain fatty acids in appetite regulation and energy homeostasis. Int J Obes.

[56] Yan S, Shi R, Li L, Ma S, Zhang H, Ye J (2019). Mannan Oligosaccharide Suppresses Lipid Accumulation and Appetite in Western-Diet‐Induced Obese Mice Via Reshaping Gut Microbiome and Enhancing Short‐Chain Fatty Acids Production. Mol Nutr Food Res.

[57] Jiao A, Yu B, He J, Yu J, Zheng P, Luo Y, et al. Sodium acetate, propionate and butyrate reduce fat accumulation in mice via modulating appetite and relevant genes. Nutrition. 2021;87–88:111198. 10.1016/j.nut.2021.111198 33761444

[58] Borgo F, Riva A, Benetti A, Casiraghi MC, Bertelli S, Garbossa S (2017). Microbiota in anorexia nervosa: The triangle between bacterial species, metabolites and psychological tests. PLoS One.

[59] Armougom F, Henry M, Vialettes B, Raccah D, Raoult D (2009). Monitoring Bacterial Community of Human Gut Microbiota Reveals an Increase in Lactobacillus in Obese Patients and Methanogens in Anorexic Patients. PLoS One.

[60] Thompson CL, Wang B, Holmes AJ (2008). The immediate environment during postnatal development has long-term impact on gut community structure in pigs. ISME J.

[61] Holman DB, Brunelle BW, Trachsel J, Allen HK. Meta-analysis To Define a Core Microbiota in the Swine Gut. mSystems. 2017;2. doi:10.1128/msystems.00004-17. 10.1128/mSystems.00004-17 PMC5443231 28567446

[62] Hahn A, Sanyal A, Perez GF, Colberg-Poley AM, Campos J, Rose MC (2016). Different next generation sequencing platforms produce different microbial profiles and diversity in cystic fibrosis sputum. J Microbiol Methods.

[63] Rintala A, Pietilä S, Munukka E, Eerola E, Pursiheimo JP, Laiho A (2017). Gut microbiota analysis results are highly dependent on the 16s rRNA gene target region, whereas the impact of DNA extraction is minor. J Biomol Tech.

