# Supplementary material for: Exploring the role of gut microbiota in host feeding behavior among breeds in swine
Source: BMC Microbiol. 2022 Jan 3;22:1. doi: 10.1186/s12866-021-02409-6 (PMC8722167; doi:10.1186/s12866-021-02409-6)

Additional file 2. Distribution of animals for three breeds in each room. The y-axis represents the number of animals from each breed group kept in each room. The x-axis represents the room (n=8). Colors represent three breeds: Duroc (green), Landrace (orange), and Large White (purple).

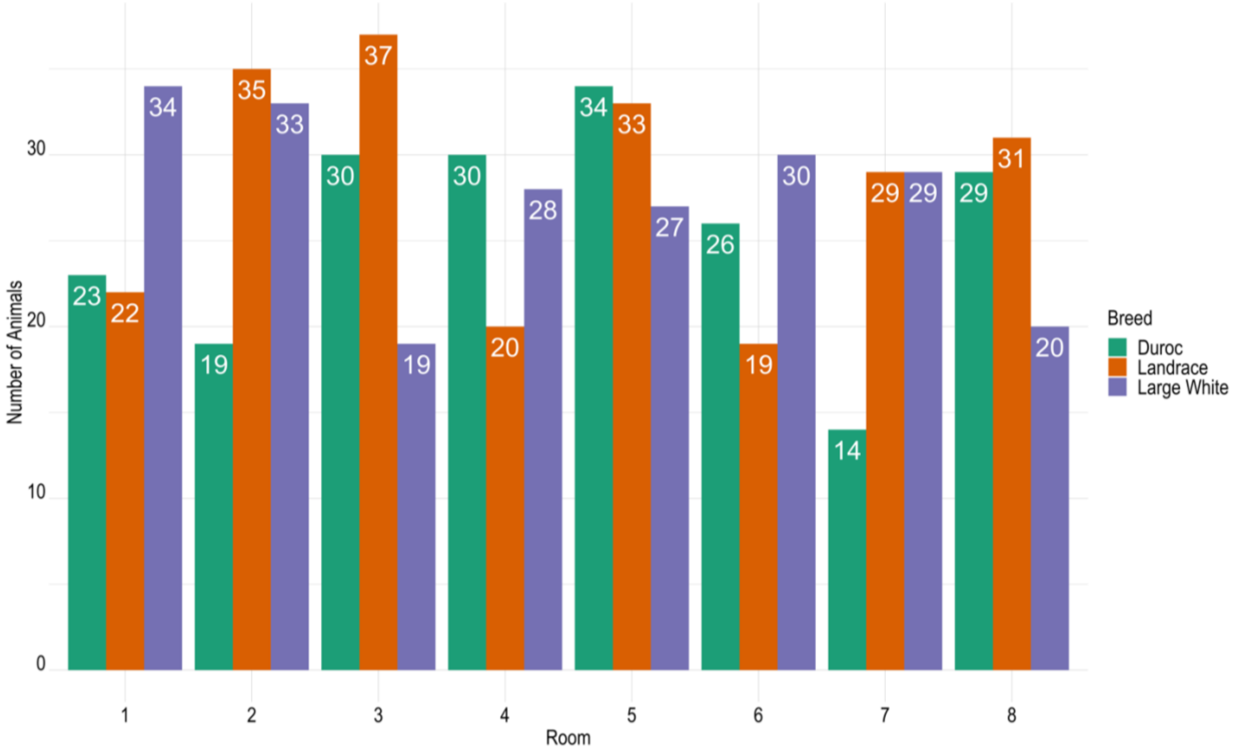

Supplement: Supplementary file 2 — Additional file 2. Distribution of animals for three breeds kept in each room. The y-axis represents the number of animals from each breed group kept in each room. The x-axis represents the room (n=8). Colors represent three breeds: Duroc (green), Landrace (orange), and Large White (purple). [file 12866_2021_2409_MOESM2_ESM.pdf]
