# Supplementary material for: Exploring the role of gut microbiota in host feeding behavior among breeds in swine
Source: BMC Microbiol. 2022 Jan 3;22:1. doi: 10.1186/s12866-021-02409-6 (PMC8722167; doi:10.1186/s12866-021-02409-6)
Supplement: Supplementary file 3 — Additional file 3. Ingredient composition for the standard diet for pigs during the present study period. The diet ingredients listed in the table were used to feed all the animals in the present study throughout the experimental period (73 to 163 days of age). This diet was provided as of 70 days of age until pigs reached the final body weight (127 kg). [file 12866_2021_2409_MOESM3_ESM.pdf]

Additional file 3. Ingredient composition for the standard diet for pigs during the present study period.

**Pig Feed Formula**	**Growing – Finishing Period**
**Ingredients**	**Amount per 1000 kg of feed (kg)**
Corn 550/15% AF	529.5
Soybean Meal	262.5
Wheat Middlings	150.0
Fat	19.0
Salt	3.5
Limestone	17.5
Phosphate 19% Dical	13.5
Lysine HCl 98%	0.6
Vitamin Alpha NGF PX	1.0
Methionine dl 99%	0.3
Choline Cl dry 60%	0.5
Hostazym	0.1
Copper sulfate	0.5
Vitamin Omega NGF PX	1.5
**Total:**	**1000**

The diet ingredients listed in the table were used to feed all the animals in the present study throughout the feeding period (73 to 163 days of age). This diet starts at 70 days of age until pigs reach the final body weight (127 kg).
