# Supplementary material for: Exploring the role of gut microbiota in host feeding behavior among breeds in swine
Source: BMC Microbiol. 2022 Jan 3;22:1. doi: 10.1186/s12866-021-02409-6 (PMC8722167; doi:10.1186/s12866-021-02409-6)

Additional file 4. Contrasts of estimates for feeding behavior traits among rooms during three time periods. Data are presented as least squares mean with the confidence interval. Colors represent different rooms (n = 8).

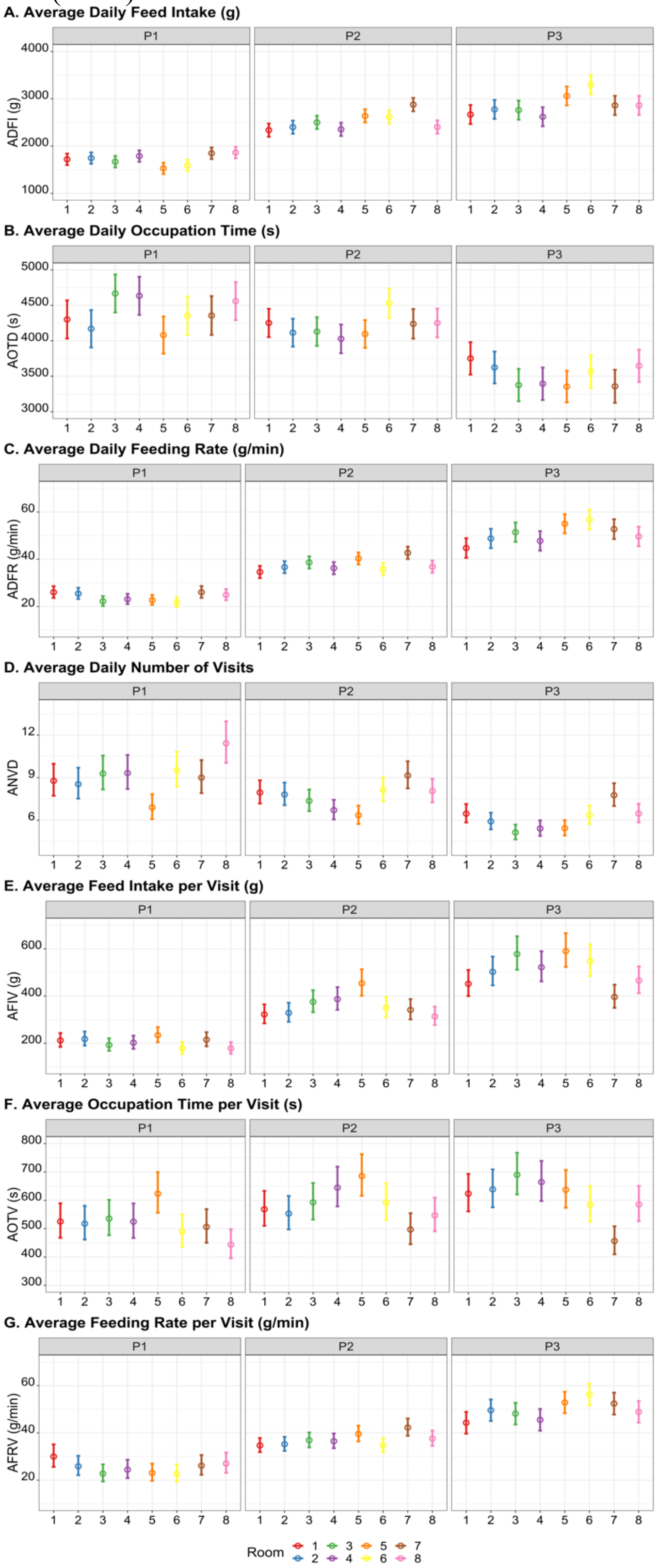

Supplement: Supplementary file 4 — Additional file 4. Contrasts of estimates for feeding behavior traits among rooms during three time periods.Data are presented as least squares mean with the confidence interval. Colors represent different rooms (n = 8). [file 12866_2021_2409_MOESM4_ESM.pdf]
