# Supplementary material for: Exploring the role of gut microbiota in host feeding behavior among breeds in swine
Source: BMC Microbiol. 2022 Jan 3;22:1. doi: 10.1186/s12866-021-02409-6 (PMC8722167; doi:10.1186/s12866-021-02409-6)
Supplement: Supplementary file 5 — Additional file 5. Summary of F values and P-values from ANOVA of fixed effects on feeding behavior traits. F values are calculated as the Mean Square Model divided by the Mean Square Residual in the ANOVA test from SAS. ADFI = average daily amount of feed consumed (g); AOTD = average daily feeder occupation time (s); ADFR = average daily feeding rate (g/min); ANVD = average daily number of visits to feeder; AFIV = average amount of feed consumed (g) per visit; AOTV = average feeder occupation time (s) per visit; AFRV = average feeding rate (g/min) per visit. * P <.05; **P < .01; ***P < .001. [file 12866_2021_2409_MOESM5_ESM.pdf]

Additional file 5. Summary of F values[1] and P-values from ANOVA of fixed effects on feeding behavior traits.

Trait[a]	Fixed Effect					
	P1		P2		P3	
	Breed	Room	Breed	Room	Breed	Room
ADFI (g)	1.50	3.78***	3.47*	6.88***	2.62	4.71***
AOTD (s)	14.54***	2.49*	21.37***	2.46*	8.75***	1.89
ADFR (g/min)	13.74***	2.37*	5.58**	4.18**	1.53	3.52**
ANVD	10.66***	4.18***	17.27***	5.04***	20.12***	6.80***
AFIV (g)	8.11***	2.05*	14.61***	3.57***	13.04***	4.16***
AOTV (s)	19.57***	2.73**	13.63***	2.25*	10.90***	3.65***
AFRV (g/min)	5.95**	1.48	2.47	2.51*	0.19	2.89**

[1] F values are calculated as the Mean Square Model divided by the Mean Square Residual in the ANOVA test from SAS.

[a] ADFI = average amount of feed consumed (g) daily during the test period; AOTD = average daily occupation time (s); ADFR = average daily feeding rate (g/min); ANVD = average daily number of visits to feeder; AFIV = average amount of feed consumed (g) per visit across testing period; AOTV = average occupation time (s) per visit across testing period; AFRV = average feeding rate (g/min) per visit across testing period.

* $P < .05$; ** $P < .01$; *** $P < .001$.
