# Supplementary material for: Exploring the role of gut microbiota in host feeding behavior among breeds in swine
Source: BMC Microbiol. 2022 Jan 3;22:1. doi: 10.1186/s12866-021-02409-6 (PMC8722167; doi:10.1186/s12866-021-02409-6)
Supplement: Supplementary file 6 — Additional file 6. Comparisons of least squares means of microbiability across breeds, time points, andfeeding behavior traits. DR = Duroc (n = 205); LR = Landrace (n = 226); LW =Large White (n = 220). T1 = 73 days of age; T2 = 123 days of age; T3 = 158 daysof age. ADFI = average amount of feed consumed (g) daily; AOTD = average daily feeder occupation time (s); ADFR = average daily feeding rate (g/min); ANVD = average daily number of visits to feeder; AFIV = average amount of feed consumed (g) per visit; AOTV = average feeder occupation time (s) per visit; AFRV = average feeding rate (g/min) per visit. Different letters a, b, and c denote P <0.05 between means in the row. [file 12866_2021_2409_MOESM6_ESM.pdf]

Additional file 6. Comparisons of least squares means of microbiability across breeds, time points, and feeding behavior traits.

Table I

Statistics	Breed*		
	**DR**	**LR**	**LW**
Least Squares Means	0.18[a]	0.16[ab]	0.14[bc]
95% Confidence Interval	0.16 - 0.19	0.14 - 0.18	0.13 - 0.16

* DR = Duroc (n = 205); LR = Landrace (n = 226); LW = Large White (n = 220).
Different letters a, b, and c denote P < 0.05 between means in the row.

Table II

Statistics	Sampling Time Point*		
	**T1**	**T2**	**T3**
Least Squares Means	0.14[a]	0.16[ab]	0.18[bc]
95% Confidence Interval	0.13 - 0.16	0.14 - 0.18	0.16 - 0.19

* T1 = 73 days of age; T2 = 123 days of age; T3 = 158 days of age.
Different letters a, b, and c denote P < 0.05 between means in the row.

Table III

Statistics	Feeding Behavior Trait*						
	**ADFI**	**AOTD**	**ADFR**	**ANVD**	**AFIV**	**AOTV**	**AFRV**
Least Squares Means	0.22[a]	0.17[b]	0.14[b]	0.16[b]	0.17[b]	0.14[b]	0.12[b]
95% Confidence Interval	0.20 - 0.25	0.14 - 0.19	0.11 - 0.16	0.14 - 0.19	0.14 - 0.19	0.12 - 0.17	0.10 - 0.15

* ADFI = average amount of feed consumed (g) daily during the test period; AOTD = average daily occupation time (s); ADFR = average daily feeding rate (g/min); ANVD = average daily number of visits to feeder; AFIV = average amount of feed consumed (g) per visit across testing period; AOTV = average occupation time (s) per visit across testing period; AFRV = average feeding rate (g/min) per visit across testing period.
Different letters a, b, and c denote P < 0.05 between means in the row.
