# Supplementary material for: Exploring the role of gut microbiota in host feeding behavior among breeds in swine
Source: BMC Microbiol. 2022 Jan 3;22:1. doi: 10.1186/s12866-021-02409-6 (PMC8722167; doi:10.1186/s12866-021-02409-6)

Additional file 7. Proportions of variance component estimates for feeding behavior traits at three time points. The plot depicts the proportions of total phenotypic variance explained by pen and sire in Null model and pen, sire, and gut microbiota in MBLUP model for Duroc (DR), Landrace (LR), and Large White (LW) at 73 days (T1), 123 days (T2), and 158 days (T3). P = the proportion of variance explained by pen effect; S = the proportion of variance explained by sire effect; M = the proportion of variance explained by the gut microbial composition; R = residual.

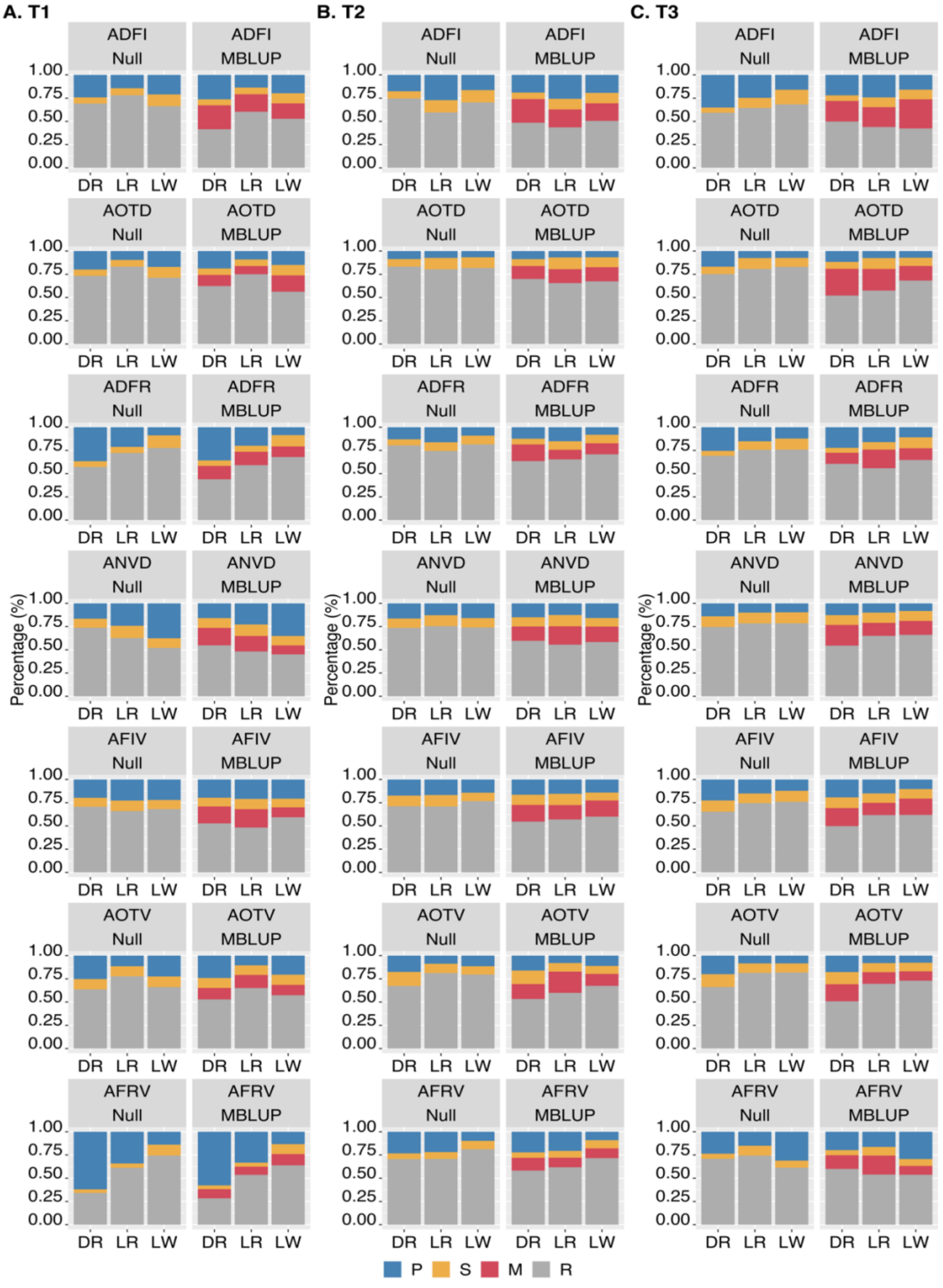

Supplement: Supplementary file 7 — Additional file 7. Proportions of variance component estimates for feeding behavior traits at three timepoints. The plot depicts the proportions of total phenotypic variance explained by pen and sire in Null model and pen, sire, and gut microbiota in MBLUP model for Duroc (DR), Landrace (LR), and Large White (LW) at 73 days (T1), 123 days (T2),and 158 days (T3). P = the proportion of variance explained by pen effect; S =the proportion of variance explained by sire effect; M = the proportion of variance explained by the gut microbial composition; R = residual. [file 12866_2021_2409_MOESM7_ESM.pdf]
