# Supplementary material for: Exploring the role of gut microbiota in host feeding behavior among breeds in swine
Source: BMC Microbiol. 2022 Jan 3;22:1. doi: 10.1186/s12866-021-02409-6 (PMC8722167; doi:10.1186/s12866-021-02409-6)

**Additional file 8.** Coefficients of identified ASVs significantly associated with feeding behavior traits at three time points. The plot depicts coefficients of the most significant ASV (least FDR) for each feeding behavior trait by breed at 73 days (T1), 123 days (T2), and 158 days (T3). Data are presented as estimated coefficients with a 95% confidence interval. Each ASV is represented using the combination of phylum, genus, and ASV number.

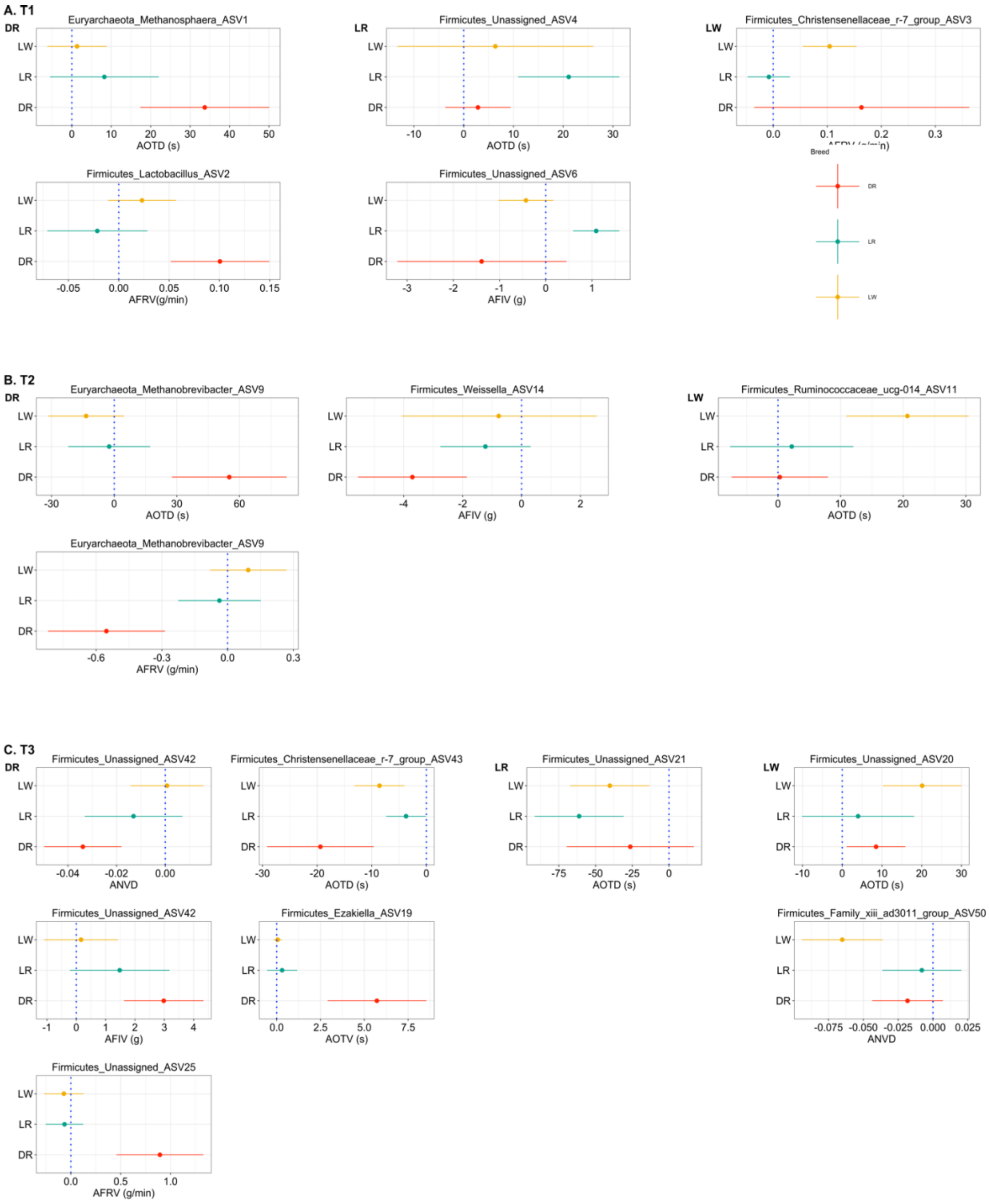

Supplement: Supplementary file 8 — Additional file 8. Coefficients of identified ASVs significantly associated with feeding behavior traits at three time points. The plot depicts coefficients of the most significant ASV (least FDR) for each feeding behavior trait by breed at 73 days (T1), 123 days(T2), and 158 days (T3). Data are presented as estimated coefficients with a 95% confidence interval. Each ASV is represented using the combination of phylum, genus, and ASV number. [file 12866_2021_2409_MOESM8_ESM.pdf]
